# Acupuncture and clomiphene for Chinese women with polycystic ovary syndrome (PCOSAct): statistical analysis approach with the revision and explanation

**DOI:** 10.1186/s13063-018-2942-7

**Published:** 2018-11-01

**Authors:** Hong-Li Ma, Liang-Zhen Xie, Jing-Shu Gao, Jing Cong, Ying-Ying Deng, Ernest H. Y. Ng, Jian-Ping Liu, Xiao-Ke Wu

**Affiliations:** 1 Department of Obstetrics and Gynecology, First Affiliated Hospital, Heilongjiang University of Chinese Medicine, Harbin, 150040 China; 2 Department of Obstetrics and Gynecology, The University of Hong Kong, Queen Mary Hospital, Pokfulam, Hong Kong; 3 Centre for Evidence-Based Chinese Medicine, Beijing University of Chinese Medicine, Beijing, 100029 China

**Keywords:** Infertility, Factorial design, Polycystic ovary syndrome, Statistical analysis approach

## Abstract

**Background:**

Polycystic ovary syndrome (PCOS) is the most common endocrinopathy of reproductive-aged women. Clomiphene is regarded as the first-line medical treatment for ovulation induction in PCOS patients and acupuncture is often used as an alternative and complementary treatment for fertility issues such as those associated with PCOS. The efficacy of acupuncture alone or combined with clomiphene still lacks strong supporting evidence. Factorial 2 × 2 designs can be used for the evaluations of two treatments within a single study, to test the main effects of acupuncture and clomiphene and their interactions.

**Methods:**

PCOSAct was designed to test the effect of clomiphene and acupuncture by three two-group comparisons in the original protocol. However, the trial was designed as a standard factorial trial and the factorial analysis approach for analyzing the data that were actually obtained during the trial was found to be more appropriate and more powerful than the three two-group comparisons described in the original protocol, so the statistical analysis approach and different datasets of PCOSAct in the primary publication were accordingly changed.

**Discussion:**

Although the statistical analysis approach used in the primary publication deviated from the statistical analysis planned in the study protocol, focusing on the main effects of the two interventions and their interactions was a more standard approach to a factorial trial and proved to be more suitable and consistent with the characteristics of the trial data. Statistically, the revision is more powerful and precise and should be more useful to the journal and the readers.

**Trial registration:**

Chinese clinical trial registry, ChiCTR-TRC-12002081. Registered on 20 March 2012. Clinicaltrials.gov, NCT01573858. Registered on 4 April 2012.

## Background

Polycystic ovary syndrome (PCOS) is the most common endocrinopathy of reproductive-aged women and it presents with a variety of features such as hyperandrogenism, oligo-ovulation with associated oligomenorrhea, obesity, acne, polycystic ovaries on ultrasonography, and infertility [1, 2]. Currently the first-line medical treatment for ovulation induction in PCOS patients is clomiphene, a non-steroidal selective estrogen receptor modulator; however, ovulation is not achieved in almost 20–40% of PCOS patients who are clomiphene-resistant [3, 4]. Acupuncture is an integral part of traditional Chinese medicine, which dates back more than 3000 years [5], and is growing in popularity among western countries; acupuncture is often used as a treatment for fertility issues such as those associated with PCOS. Clomiphene and acupuncture alone or in combination are commonly used in clinical practices, but the efficacy of acupuncture still lacks strong supporting evidence [6].

Most randomized controlled trials (RCTs) are simple two-armed designs with a single intervention compared to a single control group or are looking at two new treatments and possibly their combination within a single study [7]; RCTs are considered the gold standard for definitive evaluations of new interventions. In empirical practice, two interventions are complementary and alternative to each other to get better treatment effects. It is possible to evaluate two treatments in a single RCT. A popular design and hypothesis is the 2 × 2 factorial trial.

Factorial 2 × 2 designs can be used to combine the evaluations of two treatments within a single study [8, 9]. The standard analysis approach is then based on a factorial analysis that evaluates both treatments by pooling data with the other treatment to determine whether the effect of one treatment changes in the presence or absence of the other treatment [10]. Such 2 × 2 factorial designs are suitable in complementary alternative medicine studies to determine whether traditional Chinese medicine and western medicine have interactions, synergies, or antagonistic effects.

## Trial overview

The PCOS Acupuncture and Clomiphene Trial (PCOSAct) was a randomized, multicenter, clinical trial. The original protocol for the trial was published in 2013 [11] and the primary paper was published in *JAMA* in 2017 [12]. The PCOSAct was approved by the Ethics Committee of the First Affiliated Hospital, Heilongjiang University of Chinese Medicine (2010HZYLL-010), on 15 December 2011 and was then individually approved by local ethics committees in the participating sites. Some hospitals and local sites issued a statement that accepted the ethics approval of the First Affiliated Hospital, Heilongjiang University of Chinese Medicine, because they only had GCP ethics committees in their own hospitals (Table 1). The trial was first registered in the Chinese clinical trial registry on 20 March 2012 (ChiCTR-TRC-12002081) and then subsequently registered at ClinicalTrials.gov with identifier NCT01573858 on 10 April 2012. The first participant was enrolled on 6 July 2012 and the last participant was enrolled on 18 November 2014. The last live birth occurred on 7 October 2015. The trial lasted 39 months; 4645 patients were screened, a total of 1000 women with PCOS according to the modified Rotterdam criteria [1, 13] were enrolled in the study, and 926 completed the trial.

**Table 1:** Details of participating sites and Institutional Review Boards

Site	Hospital name	Institutional Review Board	Principal investigator
1.	Department of Obstetrics and Gynecology, First Affiliated Hospital, Heilongjiang University of Chinese Medicine, Harbin, Heilongjiang Province	Ethics Committee of First Affiliated Hospital, Heilongjiang University of Chinese Medicine (2010HZYLL-010)	Professor LH Hou
	Department of Obstetrics and Gynecology, Second Affiliated Hospital, Heilongjiang University of Chinese Medicine, Harbin, Heilongjiang Province	Accepted the ethics approval of First Affiliated Hospital, Heilongjiang University of Chinese Medicine	Professor HF Cong
2.	Centre for Reproductive Medicine, Yuhuangding Hospital, Yantai, Shandong Province	Accepted the ethics approval of First Affiliated Hospital, Heilongjiang University of Chinese Medicine	Professor CF Hao
3.	Centre for Reproductive Medicine, Daqing Oilfield General Hospital, Daqing, Heilongjiang Province	Accepted the ethics approval of First Affiliated Hospital, Heilongjiang University of Chinese Medicine	Professor YQ Gao
4.	Department of Obstetrics and Gynecology, Daqing Longnan Hospital, Daqing, Heilongjiang Province	Accepted the ethics approval of First Affiliated Hospital, Heilongjiang University of Chinese Medicine	Professor SM Du
5.	Department of Obstetrics and Gynecology, First Affiliated Hospital, Liaoning University of Chinese Medicine, Shenyang, Liaoning Province	Ethics Committee of Affiliated Hospital Liaoning University of Chinese Medicine (2012CS(KT)-003–01)	Professor X Wang
6.	Department of Gynecology, First Teaching Hospital of Tianjin University of Traditional Chinese Medicine, Tianjin	Accepted the ethics approval of First Affiliated Hospital, Heilongjiang University of Chinese Medicine	Dr Y Xia
7.	Department of Infertility, Tanggu District Maternal and Children’s Hospital, Tianjin	Accepted the ethics approval of First Affiliated Hospital, Heilongjiang University of Chinese Medicine	Professor J Ge
8.	Department of Gynecology, Wenzhou City Hospital of Chinese Medicine, Wenzhou, Zhengjiang Province	Ethics Committee of Wenzhou Chinese Medicine Hospital	Professor Y Sun
	Wenzhou Zhongshan Hospital, Wenzhou, Zhengjiang Province	Accepted the ethics approval of First Affiliated Hospital, Heilongjiang University of Chinese Medicine	Professor CL Zhu
9.	Centre for Reproductive Medicine, Zhejiang Province Hospital of Integrative Medicine, Hangzhou, Zhejiang Province	Accepted the ethics approval of First Affiliated Hospital, Heilongjiang University of Chinese Medicine	Professor CF Ding
	Department of Gynecology, Hangzhou City Hospital of Chinese Medicine, Hangzhou, Zhejiang Province	Ethics Committee of Hangzhou Hospital of Chinese Medicine (2012LL011)	Professor P Fu
10.	Department of Obstetrics and Gynecology, Shanxi Province Hospital of Chinese Medicine, Taiyuan, Shanxi Province	Accepted the ethics approval of First Affiliated Hospital, Heilongjiang University of Chinese Medicine	Professor JF Zhang
11.	Henan Province Hospital of Chinese Medicine, Zhengzhou, Henan Province	Accepted the ethics approval of First Affiliated Hospital, Heilongjiang University of Chinese Medicine	Professor JY Fu
12.	Outpatient Department, Xuzhou Maternal and Children’s Hospital, Xuzhou, Jiangsu Province	Accepted the ethics approval of First Affiliated Hospital, Heilongjiang University of Chinese Medicine	Professor ZX Hu
	Xuzhou City Hospital of Chinese Medicine, Xuzhou, Jiangsu Province	Accepted the ethics approval of First Affiliated Hospital, Heilongjiang University of Chinese Medicine	Professor PA Wang
13.	Centre for Reproductive Medicine, Huaian Maternal and Children’s Hospital, Huaian, Jiangsu Province	Accepted the ethics approval of First Affiliated Hospital, Heilongjiang University of Chinese Medicine	Professor HY Xue
14.	Department of Gynecology, Suzhou City Hospital of Chinese Medicine, Suzhou, Jiangsu Province	Ethics Committee of Suzhou Hospital of Chinese Medicine (2012LYP-02)	Professor XF Xu
15.	Institute of Integrated Traditional and Western Medicine, Tongji Hospital, Medical College, Huazhong University of Science and Technology, Wuhan, Hubei province	Accepted the ethics approval of First Affiliated Hospital, Heilongjiang University of Chinese Medicine	Dr DM Huang
	Department of Obstetrics and Gynecology, Hubei Province Hospital of Chinese Medicine, Wuhan, Hubei Province	Ethics Committee of Hubei Province Hospital of Chinese Medicine (SL2012-C022–01)	Professor ZM Zhou
16.	Centre for Reproductive Medicine, Dalian Maternal and Children’s Centre, Dalian, Liaoning Province	Ethics Committee of Dalian Maternal and Child Health Hospital	Professor XG Shao
17.	Department of Gynecology, Second Hospital, Jiangxi University of Chinese Medicine, Nanchang, Jiangxi Province	Accepted the ethics approval of First Affiliated Hospital, Heilongjiang University of Chinese Medicine	Professor RN Liang
18.	Department of Obstetrics and Gynecology, First Affiliated Hospital, Hunan University of Chinese Medicine, Changsha, Hunan Province	Ethics Committee of First Affiliated Hospital of Hunan University of Chinese Medicine (HN-LL-KY-2012-29)	Professor ZY Tan
19.	Department of Chinese Medicine, First Affiliated Hospital, Guangzhou Medical University, Guangzhou, Guangdong Province	Accepted the ethics approval of First Affiliated Hospital, Heilongjiang University of Chinese Medicine	Dr HX Ma
20.	Department of Traditional Technology, Guangdong Province Hospital of Chinese Medicine, Guangzhou, Guangdong Province	Ethics Committee of Guangdong Province Hospital of Chinese Medicine (B2012-44-01)	Professor XH Chen
	Department of Infertility, Liwan District Hospital of Chinese Medicine, Guangzhou, Guangdong Province	Accepted the ethics approval of First Affiliated Hospital, Heilongjiang University of Chinese Medicine	Professor HW Yang
21.	Department of Obstetrics and Gynecology, Affiliated Hospital, Anhui University of Chinese Medicine, Hefei, Anhui Province	Ethics Committee of First Affiliated Hospital, Anhui University of Chinese Medicine (2012AH-036-01)	Professor WL Li

The statistical analysis plan (Version 3.0) was finalized and approved on 9 April 2017. The first version of SAP was drafted in 2015; it has been written based on information contained in the study protocol version 8, dated 14 April 2012. A detailed SAP revision history is shown in Table 2. All the updated versions had been signed and dated with the signature of the person writing the SAP, approved by the SAP and Chief investigator.

**Table 2:** SAP revision history

Protocol version	Updated SAP version no.	Section number changed	Description of and reason for change	Date changed
1.0	2.0	Flow chart	Deleted the clinical outcomes from CONSORT flow diagram	28 July 2016
2.0	3.0	Dataset and statistic method	Three two-group comparisons were replaced by factorial analysis	09 April 2017

## Objectives

The PCOSAct was designed to test the effect of clomiphene and acupuncture. In the original protocol, we hypothesized that true acupuncture + clomiphene (Group A) is more likely to result in live birth than placebo acupuncture combined with clomiphene (Group B), that placebo acupuncture + clomiphene (Group B) is more likely to result in live birth than real acupuncture + placebo clomiphene (Group C), and that real acupuncture + placebo clomiphene (Group C) is more likely to result in live birth than placebo acupuncture + placebo clomiphene (Group D). However, during the revision of the primary manuscript, it became clear that the statistical approaches in the published protocol were not suitable for analyzing the data that were actually obtained during the trial.

PCOSAct was a standard factorial trial and the factorial analysis approach was found to be more appropriate and more powerful; the effectiveness of clomiphene was considered already well established due to its wide acceptance as a first-line therapy for treating infertility in PCOS patients. Therefore, the objective of the PCOSAct was preferred to the assessment of whether active acupuncture, either alone or combined with clomiphene, increases the likelihood of live births among Chinese women with PCOS. Thus, revisions of the statistical analysis approach and datasets were discussed several times among the authors, statistical specialists, and reviewers until consensus was reached. The present paper describes the differences between the initial trial protocol design and the final published paper and provides a more detailed explanation of the trial outcomes.

## Methods /Design

### Data management

Printed and electronic case report forms (CRFs) were used in the PCOSAct. Research associates were asked to fill in the CRFs at each patient meeting and the data were entered into the web-based data management system (Clinical Trial Management Public Platform, available at: http://www.medresman.org/login.aspx) within 15 days. Quality control of the data was handled at five levels.

First, the research associates were asked to fill in the CRFs quickly, accurately, completely, and truthfully; logical values and ranges in the entered data were automatically checked by the web-based data entry system. Second, data checking was performed remotely by the Data Coordination Committee staff (chaired by professor Zhang from Yale University) every month. Third, on-site supervisions were performed twice every year to identify errors in the CRFs. Fourth, the data entry staff checked the printed and electronic CRFs at the end of the treatment cycles to make sure that all of the data were the same. Fifth, the data manager performed a total data audit before the database was locked.

Intervention adherence and protocol deviations were defined. Clomiphene compliance was assessed based on the percent of individuals taking the scheduled number of pills. It was defined as: % clomiphene compliance = (number of pills taken / number of pills supposed to be taken) * 100%. % acupuncture compliance = (number of acupuncture taken / number of acupuncture supposed to be taken). The number of clomiphene supposed to be taken was calculated as the cycles of treatment multiplied by 1 or 2 or 3, depending on their treatment response. The number of acupuncture supposed to be taken was calculated as the weeks of treatment multiplied by 2. Descriptive statistics on the percent compliance (N, mean, SD, median, minimum, maximum) was summarized by randomization arm. The following were pre-defined major protocol violations with a direct bearing on the primary outcome:

1. Not meeting the diagnosis of PCOS or had regular menstrual cycles;
2. Clomiphene compliance rate was < 75% or > 125%;
3. Interval of acupuncture was more than one month;
4. Concurrent medications (clomiphene or hCG) which may impact the primary outcome.

### Sample size calculation

Due to a lack of strong preliminary data for the effectiveness of acupuncture, we chose a 10% live birth rate during the four-month period of the PCOSAct interventions as the minimal clinically detectable difference that was likely to change clinical practice. Assuming a 25% live birth rate when both interventions were active (Group A), a 15% live birth rate when one intervention was active and one was a control (Group B and Group C), and a 5% live birth rate when both interventions were controls (Group D), an 80% power at a significance level of 0.05, and a 10% drop-out rate, 1000 patients were enrolled with an allocation ratio to the four treatment arms of 1:1:1:1. All calculations were performed in SAS version 9.3 software. Original sample size calculations were based on pairwise comparisons, but they offered sufficient power and precision to clear presentation of the results with currently factorial analysis.

The statistician proposed not to do an interim analysis in this trial. The final data analysis was completed after all live birth data were obtained in the trial. Unblinding of individual study participants did not take place until all individuals had delivered and reported outcomes to the DCC.

### Definition of the outcome measures

#### Primary outcome

The primary outcome was live birth, which was measured as the proportion of participants with live birth among all participants in the primary analysis. Live birth was defined as a delivery after 20 completed weeks of gestational age as stipulated by the Improving the Reporting of Clinical Trials of Infertility Treatments (IMPRINT)-Harbin Consensus [14].

#### Secondary outcomes

The secondary outcomes included conception, pregnancy, pregnancy loss, ovulation per cycle and per participant, changes in hormonal/metabolic parameters, changes in quality of life, changes in symptoms of anxiety and depression, changes in acupuncture credibility assessments, and adverse events (AEs). Definitions of clinical reporting outcomes refer to the Harbin Consensus [14] and the International Committee for Monitoring Assisted Reproductive Technology (ICMART)–World Health Organization definitions [15].

##### Conception

Conception was defined as positive serum human chorionic gonadotropin (hCG) according to the normal range at the local site; thus, the cumulative conception rate was the proportion of participants with conception diagnosis during the four treatment cycles among all participants in the primary analysis.

##### Pregnancy

Pregnancy was defined as one or more intrauterine gestational sacs containing a fetus with positive fetal pulsation found on pelvic scanning. The cumulative pregnancy rate was the proportion of pregnant women among all participants in the primary analysis.

##### Pregnancy loss

Pregnancy loss was defined as pregnancy loss occurring from conception to 27 completed weeks of gestational age in this trial, including both biochemical pregnancy and ectopic pregnancy. The pregnancy loss rate was the proportion of women who aborted during the four treatment cycles among all women who conceived. The pregnancy loss rate was separated into pregnancy loss in the first trimester or in the second or third trimester.

##### Ovulation

Progesterone was measured every week to determine whether the women ovulated during the trial; the patient was defined as having ovulated if she had an elevated progesterone level once every four weeks. The ovulation rate per participant was calculated as the proportion of women who had ovulated at least once during the four months of treatment among the total women in the primary analysis. The ovulation rate per cycle was calculated as the proportion of cycles in which ovulation occurred among all tested cycles in the primary analysis. Conception, singleton pregnancy, and singleton live birth rate were counted separately among all ovulated cycles and among all women who ovulated.

##### Adverse events

Both serious adverse events (SAEs) and AEs were reported from different periods in time. The first included all SAEs and AEs occurring in patients before conception. For those women who conceived, SAEs and AEs were reported as those occurring in the first trimester, those occurring in the second or third trimester, and those occurring during delivery or postpartum. For fetuses and newborns, SAEs and AEs were reported as those occurring after 20 weeks of gestation through birth and those occurring within 28 days after birth, respectively.

#### Baseline characteristics

1. Demographic characteristics: age of women, age of husbands, height, weight, body mass index, hip circumference, waist circumference, waist/hip ratio, Ferriman–Gallwey score, acne score, acanthosis nigricans score, systolic blood pressure, diastolic blood pressure, mean arterial pressure, pulse pressure.
2. Transvaginal ultrasound results: uterine volume, endometrial thickness, volume of the left ovary, left ovary morphology, antral follicle count of left ovary, volume of the right ovary, right ovary morphology, antral follicle count of the right ovary, volume of both ovaries, and total antral follicle count.
3. General history: thyroid diseases, adrenal diseases, fatty liver, coronary heart disease, cerebrovascular diseases, adrenal or ovarian secretion of androgen tumor, Cushing syndrome, cancer and surgery of the stomach, uterus, cervix, or ovaries.
4. Menstrual history and pregnancy history: menstrual history included frequency of periods, menstrual cycle, and age of menarche; pregnancy history included previous pregnancies, delivery, spontaneous abortion, abortion, premature delivery, ectopic pregnancy, and length of time woman had been attempting conception.
5. Family history: diabetes, hypertension, genital system neoplasms, oligomenorrhea or amenorrhea, and pregnancy complication.
6. Lifestyle: exercise, smoking, current smoking, amount of smoking, former drinking, current drinking, how long is the drinking, the same questions of smoking and drinking to her partner, religion, and living conditions.
7. History of relative treatment: previous medication for PCOS, previous surgical therapy and assisted reproductive technology for PCOS, previous exposure of CC, maximum dose of CC, cycles of Keep taking CC, ovulation by using CC, previous exposure of acupuncture treatment and reasons, and medication to adjust menstrual cycle.
8. Genetic risk factors: if woman’s age > 35 years, if woman and partner or their close relative had Down’s syndrome, neural tube defect, anencephaly, hemophilia, muscular dystrophy, cystic fibrosis, a stillborn situation or diseases, mental retardation, birth defects, or any familial or chromosomal abnormalities, had stillbirth or spontaneous abortion during 3–4 months of pregnancy in previous marriage, took other drugs or stimulants after the pregnancy.

#### Hormonal/metabolic variables

The patients’ blood was collected at baseline and at the last visit after treatment and tested in the central laboratory (Heilongjiang University of Chinese Medicine laboratory). The assays include progesterone (nmol/L), free androgen (nmol/L), total testosterone (nmol/L), luteinizing hormone (mIU/mL), follicle-stimulating hormone (mIU/mL), LH/FSH, estradiol sex hormone-binding globulin (nmol/L), free androgen index, fasting insulin (pmol/L), glucose (mmol/L), HOMA-IR, high-density lipoprotein (mmol/L), low-density lipoprotein (mmol/L), cholesterol (mmol/L), triglyceride (mmol/L), apoprotein B (g/L), apoprotein A (g/L), lipoprotein (mg/L), lactic dehydrogenase (U/L), creatine phosphate kinase (U/L), creatine kinase isoenzyme (U/L), cystatin C (mg/L), β2-microglobulin (mg/L), homocysteine (umol/L), calcium (mmol/L), phosphorus (mmol/L), magnesium (mmol/L), potassium (mmol/L), sodium (mmol/L), chlorine (mmol/L), total bile acid (umol/L), total bilirubin (umol/L), direct bilirubin (umol/L), indirect bilirubin (umol/L), creatinine (umol/L), urea (mmol/L), glutamic-pyruvic transaminase (U/L), and glutamic oxalacetic transaminase (U/L).

#### Exploratory outcomes

Some clinical outcomes in the PCOSAct that were not prespecified in the protocol but merited reporting conformed to the Infertility Trial CONSORT [16] were categorized as exploratory outcomes in the primary publication. These included singleton live birth, twin live births, infant birth weight, sex ratio at birth, pregnancy duration, singleton pregnancy, time to conception, pregnancy loss in the first trimester, pregnancy loss in the second or third trimester, biochemical pregnancy, and ectopic pregnancy. The singleton live birth rate was calculated according to the number of women with single live births among total live births and the twin live birth rate was calculated as the number of women with twin live births among total live births. Pregnancy duration was calculated as the number of weeks from the first day of the last menstrual period to the time of delivery. Time to conception was defined as the number of days from the first day of taking clomiphene or placebo clomiphene to the day of a positive serum hCG test.

### Missing data imputation

The reasons for participants with no completed follow-ups were summarized. The withdrawn rates were 6.0%, 5.6%, 10.8%, and 7.2% (*P* = 0.11) in the four groups, respectively. Attempts are made to follow up all participants. Follow-up information was obtained from the patients’ friends, family members, general practitioner, or the site investigators. Missing data for primary outcomes and other clinical outcomes including conception and pregnancy in 74 women were excluded from the primary analysis. For the other secondary outcomes, we did not impute the missing data, but reported the actual sample size of each variable.

## Results

The revision of the primary manuscript published in *JAMA* lasted from February to June 2017; it was revised five times with two revisions being major revisions. During the revision process, it became clear that the original study design in the published protocol was not sufficient for analyzing the data that were actually obtained in the trial. In order to address this, significant changes were made to the statistical analysis approach and the datasets compared to the published protocol; the figures, tables, and language were heavily modified in the revisions in order to reflect these changes.

### Revision of the statistical analysis approach

**Fig. 1:**
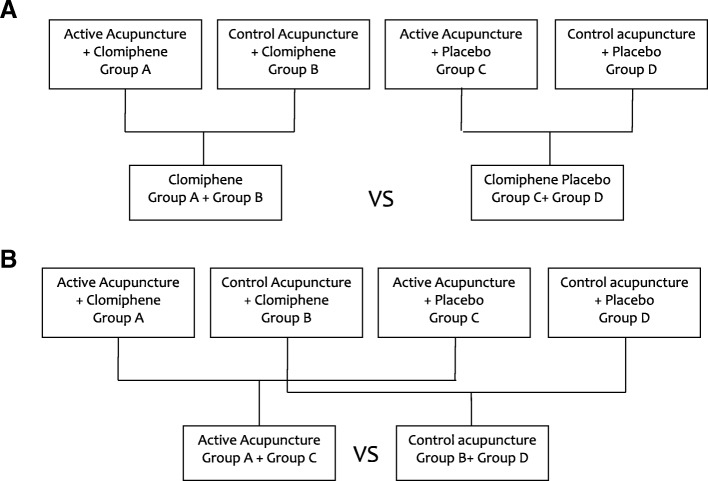
The main effect analyses of clomiphene and acupuncture. **a** The active acupuncture + clomiphene and the control acupuncture + clomiphene groups (Group A and Group B) were combined and compared with the combination of the active acupuncture + placebo and control acupuncture + placebo groups (Group C and Group D) to test the main effect of clomiphene. **b** In case of the active acupuncture main effect, the active acupuncture + clomiphene and active acupuncture + placebo groups (Group A and Group C) were compared with the combination of the control acupuncture + clomiphene and control acupuncture + placebo groups (Group B and Group D)

The three two-group comparisons that were described in the published protocol were replaced by factorial analysis in the primary publication. Logistic or linear regression analyses were used for studying the main effects of the two interventions and the interactions between the two interventions. No significant interactions were found between the two interventions, so this meant that the main effect of each treatment involved two composite comparisons. For the main effects of clomiphene and active acupuncture, detailed combinations and comparisons were shown in Fig. 1a, b. Two-sided *P* < 0.05 indicated statistical significance.

### Revision of datasets

According to the intention-to-treat (ITT) principle, all randomized women should be included in the analysis and the violation cases (or withdrawn cases) should also be included in the outcome measurements. We intended to follow the ITT principle in the analyses, but due to the editor’s and reviewers’ suggestions, the populations vary in the different tables. The baseline data followed the ITT principle and all randomized women were included in the baseline table. For the primary and secondary clinical outcomes, the total number of women in each group omitted the patients who had withdrawn. In fact, these outcomes were unavailable for patients who withdrew and the implicit assumption that no live births occurred among the withdrawn patients was implausible. The primary analysis dataset therefore excluded the patients who had withdrawn and the withdrawn rates were similar among four groups. For AEs, all women who received treatments were included in the analysis. There were in four patients in total (one patient in each group) who were randomized but did not receive any treatments in this trial.

### Revision of figures

**Fig. 2:**
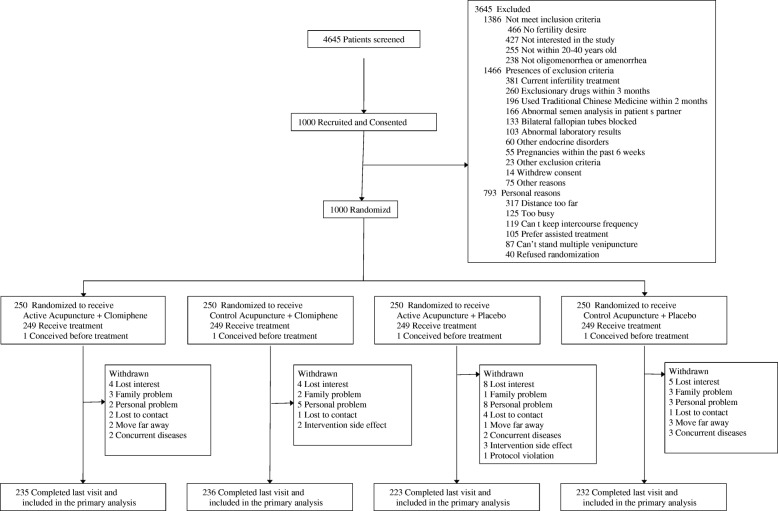
Revised CONSORT *diagram* in the PCOSAct. Adopted from *JAMA* 2017;317(24):2502–14

The first figure in the PCOSAct primary publication was the flow chart showing the Consolidated Standards of Reporting Trials (CONSORT) diagram [17]; the new version contained significant changes to the initial design and was different from similar flow charts seen in most other PCOS trials [18, 19]. The new CONSORT diagram comprised the number of people screened, eligible, consented, randomized, receiving their allocated treatment, withdrawn/lost to follow-up, and the number of women included in the primary analysis (Fig. 2). The information on conceptions, pregnancy loss, live births, and the numbers completing the trial without conceiving were deleted; the information on the number of women who completed the trial and the number of women included in the primary analysis were inserted. Following randomization, we added the number of women who received treatment as randomized and the those who did not receive treatment along with the reasons for this. The number of withdrawals and the reasons over the course of the trial were summarized by treatment arm.

The second figure in the primary publication showed the modified Kaplan–Meier curves for live births according to treatment group. Following the editor’s suggestion, data below the horizontal axis, which were the cumulative numbers of live births, were not deemed essential and so the label for the y-axis was changed from “Live births” to “Percentage of women with live births.” The format of the graph should be output directly from the software used to create it in an editable vector file format, such as .wmf or .eps, or as Excel graphs (including the linked data file).

### Revision of tables

For the baseline table, the sample size was added to the column headings in each group; for the continuous variables, the sample size was reported only when it differed from the complete sample in each group. The first column of the revised table included the variables and the explanations for how the data were expressed for each variable were included in the second column. To be able to compare changes in variables from baseline to last visit in other tables, all variables with changes in other tables such as the quality of life questionnaire and hormonal/metabolic parameters were reported in the baseline table. Also, footnotes were added describing the range of possible scores in the quality of life questionnaires, the definitions and explanations of specialized terms used in obstetrics and gynecology, and the conversion factors for converting the laboratory indexes between international units and conventional units.

The clinical outcome table was divided into two parts; although this made for a rather large table, both parts were included in a single table. The first part of the table reported outcomes by group and presented basic information about the frequency of study outcomes in each arm; the second part of the table reported the combined comparisons. The overall main effect estimates for the active acupuncture and clomiphene treatments were presented on the right half of the table. The final column included a single *p* value for the interactions. This table thus capitalized on the best features of the factorial analysis approach. The third table reported SAEs and AEs according to the randomization groups; it was also divided into two parts similar to the clinical outcome table.

## Discussion

The revised statistical analysis approach in the PCOSAct primary publication deviated from the previously published protocol because it became clear that the factorial analysis approach would enhance the accuracy and precision of the estimates of the treatment effects and would allow us to obtain the most robust conclusions because data from all four treatment groups could be used to estimate the main effects of both interventions in the statistical analysis.

The proposed study in the published protocol focused on three two-group comparisons between treatment arms, one for each hypothesis: Group A vs Group B, Group B vs Group C, and Group C vs Group D. However, the statistical specialists expressed different opinions on the hypothesis while reviewing the primary publication. Because the PCOSAct had a 2 × 2 factorial design, the three hypotheses that were originally listed in the published protocol did not take advantage of the full power of a factorial analysis and so the focus in the revision for the primary publication was placed on the main effects of the treatments and the possible interactions between them. This new hypothesis asserted that the effect of clomiphene on live birth rates depended on whether a participant received active acupuncture or control acupuncture. Or, equivalently, the effect of active acupuncture depended on whether a participant received clomiphene or placebo. There were no significant interactions observed in the factorial analysis between clomiphene and acupuncture; thus, the main effect of each treatment involved two composite comparisons.

The reasons for why we changed from three two-group comparisons in the published protocol to a factorial analysis in the primary publication were as follows.

1. *Consistency with the hypothesized live birth rates used in the sample-size calculations*. When performing the sample-size calculations for the published protocol, we assumed that the live birth rate following four treatment cycles would be 25% in the active acupuncture + clomiphene group, 15% in the control acupuncture + clomiphene group, 15% in the active acupuncture + placebo group, and 5% in the control acupuncture + placebo group. The main effect of clomiphene was hypothesized to be 10%, regardless of whether active or control acupuncture is used. Similarly, the main effect of active acupuncture was also hypothesized to be 10%, regardless of whether clomiphene or placebo is used. Thus, the hypothesized live birth rates in the original trial protocol favored the use of a factorial analysis [20].
2. *Precision of the estimates of the treatment effects*. With a factorial analysis, data from all four treatment groups were used to estimate each main effect [19–21]. In contrast, the three hypotheses in the original two-group comparisons would only have tested data from two treatment arms at a time. Thus, the factorial analysis provided more precise estimates of the main effects of clomiphene and active acupuncture.
3. *Better fit with the PCOSAct data*. The study results showed negligible interactions between the two treatments for the main outcome of live births. The results thus lent themselves nicely to estimations of more precise main effects of the treatments and arguably to a more straightforward presentation of the findings [21].

For the clinical outcomes, the information on live births and other outcomes was not available for patients who withdrew from the trial. Using the original group size (250 patients per treatment arm) as the denominator in the calculations made an implicit assumption that no live births occurred among the patients who withdrew implausible. Thus, the extreme assumption that no births occurred among any of the women who withdrew was almost certainly false and yields underestimations of the live birth rates in all four treatment arms. Thus, the total number of women for each treatment arm in terms of live birth rates and other clinical outcomes omitted patients who withdrew. The 95% confidence intervals shown in the primary publication were based on a linear probability model using robust standard errors to account for the heteroscedasticity that is intrinsic to such models. A linear probability model form was chosen to maintain fidelity with the pattern of hypothesized live birth rates specified in the published protocol, which posited additive effects for each treatment and no interaction based on the PCOSAct data. The A vs B and C vs D comparisons that were prominently featured in the second table in primary publication correspond to two of the original hypotheses specified in the published protocol.

The statistical analysis approach used in the PCOSAct primary publication deviated from the planned statistical analysis in the published study protocol because this new approach was found to be more suitable and consistent with the characteristics of the data that were actually collected during the trial. From a statistical point of view, the revisions made in the primary publication offered a more powerful and precise analysis and should thus be more acceptable and useful to the readers.

## Abbreviations

AE: Adverse event
CC: Clomiphene citrate
CONSORT: Consolidated Standards of Reporting Trials
CRF: Case report form
DCC: Data coordination committee
hCG: Human chorionic gonadotropin
ICMART: International Committee for Monitoring Assisted Reproductive Technology
IMPRINT: Improving the Reporting of Clinical Trials of Infertility Treatments
ITT: Intention to treat
PCOS: Polycystic ovary syndrome
RCT: Randomized controlled trial
SAE: Serious adverse event
SAP: Statistical analysis plan
SD: Standard deviation
